# Acceptability and Usability of Blood-Based and Oral Fluid HCV Self-Testing Among Men-Who-Have-Sex-with-Men and Transgender Women on PrEP

**DOI:** 10.1007/s10461-026-05207-1

**Published:** 2026-06-22

**Authors:** Hugo Perazzo, Sandra W. Cardoso, Thiago S. Torres, Daniel Bezerra, Ketiuce Zukeram, Marcos Benedetti, Julio Moreira, Brenda Hoagland, Cristina Pimenta, Beatriz Grinsztejn, Valdilea G. Veloso

**Affiliations:** 1 Evandro Chagas National Institute of Infectious Diseases (INI), Oswaldo Cruz Foundation (FIOCRUZ), Rio de Janeiro, RJ Brazil; 2 Grupo Arco-Íris, Rio de Janeiro, RJ Brazil

**Keywords:** Hepatitis C, Self-testing, Diagnosis, Feasibility studies

## Abstract

Hepatitis C virus self-testing (HCVST) has emerged as a potential strategy to expand testing among key populations. We assessed the feasibility of HCVST in cisgender men-who-have-sex-with-men (cis-MSM) and transgender women (TGW) on pre-exposure prophylaxis (PrEP). This cross-sectional study included cis-MSM or TGW attending a PrEP consultation in Rio de Janeiro (Brazil). Participants performed HCVST using blood-based and oral-fluid kits on the same day under observation. Difficulties, errors and assistance during HCVST were recorded. Re-reading and re-testing concordance [Kappa(k)] and values/preferences were assessed. A total of 250 participants (88% cis-MSM, age = 34 [IQR,28–41] years, 42% with high education level) were included. The main steps where participants requested assistance (95%CI) for blood-based HCVST were to add buffer [35.6%(29.9–41.8)] and to collect blood sample with the dropper [34.0%(28.4–40.1)]. The main error during oral fluid HCVST was incorrect collection of oral fluid [29.6% (95%CI,24.2–35.6)]. A total of 62.4% (95%CI,56.2–68.2) and 28.8% (95%CI,23.5–34.8) participants needed assistance in at least one step of blood-based and oral fluid HCVST, respectively. Lower education level was associated with higher odds of needing assistance for blood-based HCVST [aOR = 2.07 (95%CI,1.99–3.59),*p* = 0.009]. Re-reading and re-testing k-indexes were 0.92 and 0.89 for blood-based, and 1.00 and 0.75 for oral fluid HCVST, respectively. More than 95% of people felt safe; would repeat or would recommend HCVST. A total of 46.4% (95%CI,40.3–52.6) preferred oral fluid versus 36.4% (95%CI,30.6–42.6) who preferred blood-based. A relatively high proportion of participants needed assistance, especially for blood-based HCVST. Despite these challenges, high re-reading and re-testing agreements were observed and HCVST was well-accepted.

**Supplementary Information:**

The online version contains supplementary material available at https://doi.org/10.1007/s10461-026-05207-1.

## Introduction

The World Health Organization (WHO) estimates that 50 million people globally have hepatitis C virus (HCV) infection, with about 1.0 million new infections occurring per year [1]. People from key populations, such as gay, bisexual and other men-who-have-sex-with-men (MSM), people who inject drugs and transgender women (TGW) are at increased vulnerability for HCV infection. Moreover, TGW might have additional exposure given silicone injections [2, 3].

The HCV continuum of care remains deficient in many countries, especially due to low awareness, a moderately low level of HCV screening and to challenges/barriers from screening to HCV treatment [4]. Strategies have been proposed to increase HCV testing worldwide. The use of point-of-care tests, such as HCV antibody rapid-test and reflexive HCV-RNA testing, was associated with a 2 to 3-fold increase in the rate of HCV testing/year [5]. Moreover, electronic medical record systems to identify people at risk [6] and the use of vendors machines to distribute tests [7] might increase HCV screening. HCV self-testing (HCVST), where people can collect their own specimen and perform a test themselves, can be a strategy to scale up HCV testing [8].

HCVST has been recommended by WHO as a potential strategy to expand testing, especially among key populations. Preliminary analyses reported that universal HCVST can be cost-effective, especially in high-risk populations, and this toll can substantially expand access to testing [9]. Blood-based and oral fluid HCV self-tests kits have been developed. Evidence of feasibility of oral fluid HCVST have been reported [10]. However, data on acceptability and usability of HCVST, remain scarce in Western countries, especially among populations at increased vulnerability from the global south. Because HIV and HCV share overlapping transmission networks in this population, offering HCVST during routine pre-exposure prophylaxis (PrEP) follow-up visits could efficiently integrate hepatitis C screening into existing HIV prevention care pathways [11]. We aimed to evaluate acceptability, usability, re-reading/re-testing agreement and participant’s perspectives of HCVST using blood-based and oral fluid kits in cisgender-MSM and TGW attending a PrEP consultation in Rio de Janeiro (Brazil).

## Materials and Methods

### Ethics Statement

The study protocol was approved by the Institutional Review Board (IRB) from INI/FIOCRUZ (IRB 69747223.4.0000.5262) and from WHO’s Research Ethics Review Committee (WHO/ERC) (WHO/ERC Protocol ID: ERC.0003934). All participants signed an informed consent prior to study enrollment.

### Study Design and Population

This cross-sectional study recruited users of oral PrEP from 08-Jul-2024 to 31-Jan-2025 at a tertiary center (INI/FIOCRUZ) in Rio de Janeiro, Brazil. Cis-MSM and TGW who were attending a PrEP consultation (first consultation for PrEP start or people who were already on PrEP) provided by the Brazilian Public Health System (PrEP-SUS) were eligible. Exclusion criteria were age < 18 years or inability to consent to participate in the study.

### Pre-testing Interview

Pre-testing interview was performed by a trained nurse and included socio-demographic characteristics (sex at birth, age, self-reported skin color and gender, schooling and employment status and monthly family-income), risk-factors for HCV infection (blood transfusion, former of current injection drug use, tattoo/piercing and hemodialysis), sexual behavior in the last 6 months and previous HCV testing. Chemsex was defined as the intentional use of psychoactive substances, such as methamphetamine or mephedrone, before or during sexual activity to enhance or prolong the experience [12]. Data were entered in electronic forms of REDCap (Research Electronic Data Capture, https://www.project-redcap.org/) [13].

### HCV Self-Tests Kits

Blood-based [Wondfo HCV Self-Test^®^ (Wondfo Biotech Co, Guangzhou, China)] and oral fluid HCVST kits [OraQuick Hepatitis C Self-Test^®^ (OraSure Technologies, Bethlehem, USA)] were adapted by manufacturers from Wondfo^®^ One Step Hepatitis C Virus and from HCV OraQuick^®^ HCV Rapid Antibody Test, respectively. Both kits were repackaged for self-test use by the manufacturers and labelled “for research use only (RUO)”. English instructions for use (IFU) were translated to Portuguese and included in the package. The kits contained all accessories needed to perform HCVST. Videos describing step-by-step for HCVST with instructions to interpretation of results by both kits with audio and sub-titles in Portuguese were available in an electronic tablet for pre-testing information. The videos were produced by the research team in accordance with the manufacturers’ IFU for each test.

### HCV Self-Testing

All participants performed blood-based and oral fluid HCVST on the same day. Participants were encouraged to perform HCVST on their own without assistance using both kits while being observed by a trained healthcare worker (nurse) in a private room. Participants were free to decide to perform either blood-based or oral fluid test first during HCVST. After finishing self-testing with one HCV self-test kit, they started the procedure with the other kit. Participants were advised to avoid consuming food or using oral care products for 15 and 30 min, respectively, before oral fluid HCVST. Additionally, for oral fluid HCVST, the participants were instructed not to touch the flat pad with their fingers during sample collection, as per manufacturer instructions, to avoid contamination that could compromise test validity. The healthcare worker noted pre-testing activities, difficulties and errors during self-testing with both kits. For blood-based HCVST, the following steps were analysed by the healthcare worker: (1) performing a fingerstick, (2) collecting the blood sample with the dropper, (3) applying the sample to the specimen well, (4) adding buffer to the buffer well, (5) timekeeping, and (6) difficulty interpreting the test result. For oral fluid HCVST, the healthcare worker recorded the following steps: (1) opening the tube, (2) placing the tube in the plastic stand, (3) inserting the test cassette into the tube, (4) checking whether the participant touched the flat pad, (5) collecting oral fluid sample, (6) timekeeping, and (7) difficulty interpreting the test result. If requested by the participant, the healthcare worker could provide assistance for any step of both HCVST kits. The required assistance, as well as which step participant requested help, were recorded. HCVST results with blood-based and oral fluid kits were first interpreted and recorded by the participant, and then the same test-dispositive were interpreted by the healthcare worker. Results from HCVST were not used for clinical decision, since those kits were used as RUO.

### HCV Testing

After interpretation of HCVST with both kits, all participants were re-tested by the healthcare worker using similar blood-based and oral fluid kits. These results were read and interpreted by the healthcare worker and communicated to the participants.Although the professional-use version of the OraQuick^®^ HCV test is registered with the Brazilian National Health Authority (“*Agência Nacional de Vigilância Sanitária*”, ANVISA) and approved for clinical decision-making when administered by healthcare workers, all participants with a positive result on any screening test—whether self-administered or professionally performed, using oral fluid or blood-based—were referred for confirmatory HCV RNA testing at the study facility. This conservative protocol was implemented to maximize case detection and ensure no true positive infections were missed. People with detectable HCV-RNA were linked to HCV treatment at INI/FIOCRUZ.

### Post-testing Interview

Participants were interviewed by the healthcare worker with a structured questionnaire to assess post-self-testing perspectives and preferences. Participants’ perspectives include if he/she felt safe performing HCVST; willingness to recommend HCVST to a parent, friend, or sexual partner; disposition to repeat HCVST with blood-based and/or oral fluid HCV self-test kit. Additionally, participants were requested to categorize reading the HCVST results and the overall experience with both kits as “very easy”, “easy”, slightly difficult”, “difficult” or “very difficult”. For post-testing preferences, participants were asked about which type of HCV self-test kit did they prefer for HCV testing (blood-based vs. oral fluid vs. no preference). Moreover, they were asked where they would like to perform an HCVST in the future.

### Statistical Analysis

Categorical variables and continuous variables were reported as absolute (n)/relative frequency (%) and as median (interquartile range, IQR), respectively. Independent groups were compared using Chi-squared test for proportions and Mann-Whitney for quantitative variables. Usability of blood-based and oral fluid HCVST was assessed by proportions (95% confidence interval, CI]) of correct pre-testing activities, difficulties, errors observed, and assistance needed during HCVST. The proportion of people who needed assistance for at least one step of blood-based and oral fluid HCVST was reported. Logistic regression models were performed to assess factors associated with the need for assistance for HCVST. Variables found to be associated [p value ≤ 0.10] with the need of assistance for at least one step during HCVST were entered into a multivariate model for blood-based and other for oral fluid HCVST. The severity of multicollinearity among variables entered in each multivariate model was quantified by the variance inflation factor (VIF).

Acceptability and participants’ perspectives about HCVST were assessed by proportions (95%CI) of participants who felt safe, would recommend and/or would repeat HCVST and perceptions on overall experience of HCVST. Additionally, preferences for future HCV testing using self-tests were evaluated. The healthcare worker checked the HCV self-test cassette from both kits to assess re-reading (inter-observer) agreement blinded to the participant’s results interpretation. Re-reading agreement was defined as the concordance between the participant’s and the healthcare worker’s interpretation of the HCVST result. Re-testing agreement was determined as the concordance between the participant’s interpretation of the HCVST result and the healthcare worker’s interpretation of the professional use each test result, excluding invalid results. Both re-reading and re-testing concordance were analyzed using Cohen’s kappa (k index). Statistical analyses were conducted using Stata SE18 (64 bits) for Windows (2017; StataCorp LP, College Station, TX, USA).

## Results

A total of 250 participants [88% cis-MSM and 12% TGW; median age of 34 (IQR, 28–41) years; 77% with self-report as Mixed or Black, 42.4% with college degree or higher] were included. Of them, 59.2% self-reported previous HCV testing. Participants had low HCV risk by previous blood transfusion (*n* = 8, 3.2%) and former or current inject drug use (*n* = 8, 3.2%). On the other hand, vulnerability to HCV related to sexual behaviour was high. The median (IQR) number of sexual partners in the last 6 months prior to HCV testing was 10 (3–27). A total of 74% (*n* = 185) and 76% (*n* = 190) had unprotected insertive and receptive anal sex in the past 6 months, respectively. Moreover, 24.8% (*n* = 62) had at least one episode of chemsex in the previous 6 months. Table 1 describes the socio-demographic characteristics of enrolled participants.

**Table 1:** Characteristics of participants included in the study

	All (*n* = 250)	MSM (*n* = 219)	TGW (*n* = 31)
Male sex at birth ^a^	250 (100.0)	219 (100.0)	31 (100.0)
Age ^b^	34.1 (27.5–41.0)	35.2 (28.1–41.3)	28.9 (23.8–38.3)
*Self-reported skin colour* ^a^			
White	74 (29.6)	68 (31.1)	6 (19.4)
Black	75 (30.0)	68 (31.1)	7 (22.6)
Mixed	93 (37.2)	78 (35.6)	15 (48.4)
Other	7 (2.8)	4 (1.8)	3 (9.7)
Chose not to disclose	1 (0.4)	1 (0.5)	0 (0.0)
*Employment status* ^a^			
Formally employed	127 (50.8)	122 (55.7)	5 (16.1)
Informally employed	60 (24.0)	42 (19.2)	18 (58.1)
Unemployed	35 (14.0)	31 (14.2)	4 (12.9)
Student	26 (10.4)	22 (10.0)	4 (12.9)
Retired	1 (0.4)	1 (0.5)	0 (0.0)
Chose not to disclose	1 (0.4)	1 (0.5)	0 (0.0)
*Familly monthly income* ^a^			
None	10 (4.0)	8 (3.7)	2 (6.5)
Up to 1 minimum wage	69 (27.6)	54 (24.7)	15 (48.4)
1–2 minimum wages	55 (22.0)	48 (21.9)	7 (22.6)
2–3 minimum wages	38 (15.2)	33 (15.1)	5 (16.1)
3–4 minimum wages	20 (8.0)	19 (8.7)	1 (3.2)
4–6 minimum wages	26 (10.4)	26 (11.9)	0 (0.0)
6–10 minimum wages	24 (9.6)	23 (10.5)	1 (3.2)
> 10 minimum wages	6 (2.4)	6 (2.7)	0 (0.0)
Chose not to disclose	2 (0.8)	2 (0.9)	0 (0.0)
*Education level* ^a^			
Less than elementary school	3 (1.2)	1 (0.5)	2 (6.5)
Elementary school	20 (8.0)	12 (5.5)	8 (25.8)
High school	89 (35.6)	73 (33.3)	16 (51.6)
Technical/Vocational school	32 (12.8)	29 (13.2)	3 (9.7)
College degree or higher	106 (42.4)	104 (47.5)	2 (6.5)
Previous blood transfusion ^a^	8 (3.2)	8 (3.7)	0 (0.0)
Formal or current inject drug use ^a^	8 (3.2)	6 (2.7)	2 (6.5)
Previous injection of industrial silicone ^a^	10 (4.0)	1 (0.5)	9 (29.0)
Previous tattoo or piercing ^a^	150 (60.0)	123 (56.2)	27 (87.1)
Previous HCV testing ^a^	148 (59.2)	127 (58.0)	21 (67.7)
*Sexual behaviour in the last 6 months*			
Number of sexual partners ^b^	10.0 (3.0–27.0)	8.0 (3.0–25.0)	15.0 (6.0–35.0)
*Condomless sex intercourses* ^a^			
Never	36 (14.4)	29 (13.2)	7 (22.6)
Always	46 (18.4)	43 (19.6)	3 (9.7)
Few times	165 (66.0)	144 (65.8)	21 (67.7)
Chose not to disclose	3 (1.2)	3 (1.4)	0 (0.0)
Condomless insertive anal sex ^a^	185 (74.0)	172 (78.5)	13 (41.9)
Condomless receptive anal sex ^a^	190 (76.0)	160 (73.1)	30 (96.8)
At least one episode of chemsex ^a^	62 (24.8)	52 (23.7)	10 (32.3)

Data expressed as n (%) ^a^ or median (IQR) ^b^

*MSM* men who have sex with men, *TGW* transgender women

Minimum wage = BR$ 1,420 (US$ 258) per month

### Difficulties, Errors and Assistance Needed for Blood-Based HCVST

All 250 participants completed all steps of the blood-based HCVST. Table 2 summarize observed difficulties, errors and assistance provided during blood-based HCVST [expressed as N [% 95%CI)]. In pre-testing activities, 42.4% (*n* = 106) of participants read IFU and 50.4% (*n* = 126) watched step-by-step video before testing. Only 5.6% (*n* = 7) people did both pre-testing activities. Some participants had difficulties (95%CI) to collect blood sample with the dropper [40.0% (34.1–46.2)] and to fingerstick [21.6% (16.9–27.2)]. Errors on timekeeping for reading results (8.8%) was low. A total of 156 participants [62.4% (56.2–68.2)] requested assistance for at least one step of blood-based HCVST. The top reasons for needed assistance (95%CI) were to add buffer in the buffer well [35.6% (29.9–41.8)], to collect blood sample with the dropper [34.0% (28.4–40.1)] and to add blood sample in the specimen well [30.8% (25.4–36.8)]. Additionally, 20.8% (*n* = 52) requested assistance for fingerstick. In a sensitivity analysis, excluding the step of collecting blood sample with the dropper, the need of assistance for at least one step was 56.8% (95%CI, 50.6–62.8). A total of 4 participants [1.6% (95%CI 0.6–4.2)] could not report the result of blood-based HCVST.

**Table 2:** Healthcare provider assessment of difficulties, errors and necessity of assistance for blood-based and oral fluid HCV self-testing (HCVST) performed on the same day by MSM (*n* = 219) and TGW (*n* = 31) under PrEP

Blood-based HCVST (*n* = 250)	*n*	% (95%CI)	Oral fluid HCVST (*n* = 250)	*n*	% (95%CI)
*Pre-testing activities*			*Pre-testing activities*		
Participants who read the IFU	106	42.4 (36.4–48.6)	Participants who read the IFU	112	44.8 (38.7–51.0)
Participants who watched step-by-step video	126	50.4 (44.2–56.6)	Participants who watched step-by-step video	130	48.0 (45.8–58.2)
*Difficulties observed during HCVST*			*Difficulties observed during HCVST*		
To perform a finger-prick	54	21.6 (16.9–27.2)	To insert test cassette in the tube	14	5.6 (3.3–9.3)
To collect blood sample with the dropper	100	40.0 (34.1–46.2)			
To add blood sample in the specimen well	51	20.4 (15.8–25.9)			
*Errors observed during HCVST*			*Errors during HCVST*		
To add buffer in the buffer well	23	9.2 (6.2–13.5)	Touched the flat pad	3	1.2 (0.4–3.7)
To timekeeping for reading results	22	8.8 (5.9–13.0)	To collect oral fluid sample	74	29.6 (24.2–35.6)
			To timekeeping for reading results	21	8.4 (5.6–12.6)
*Assistance needed during HCVST*			*Assistance needed during HCVST*		
To perform a finger-prick	52	20.8 (16.2–26.3)	To open the tube	18	7.2 (4.6–11.2)
To collect blood sample with the dropper	85	34.0 (28.4–40.1)	To place the tube into the plastic stand	34	13.8 (10.0-18.7)
To add blood sample in the specimen well	77	30.8 (25.4–36.8)	To collect oral fluid sample	20	8.0 (5.2–12.1)
To add buffer in the buffer well	89	35.6 (29.9–41.8)	To place the test device in the test tube	11	4.4 (2.4–7.8)
To read HCVST result	45	18.0 (13.7–23.3)	To read HCVST result	40	16.0 (11.0-21.1)
Assistance in at least one step of HCVST	156	62.4 (56.2–68.2)	Assistance in at least one step of HCVST	72	28.8 (23.5–34.8)
Assistance in at least one step of HCVST without the use of dropper	142	56.8 (50.6–62.8)			

Data expressed as absolute number (n) and proportion (%) (95% Confidence Interval, CI)

*HCVST* hepatits C self-testing, *IFU* instructions for use, *MSM* men who have sex with men, *TGW* transgender women

### Difficulties, Errors and Assistance Needed for Oral Fluid HCVST

All 250 participants completed all steps of oral fluid HCVST. Similar to blood-based HCVST, 45% (*n* = 112) of people read IFU and 48% (*n* = 130) watched step-by-step video before testing. The main error during this type of HCVST was incorrect collection of oral fluid [*n* = 74, 29.6% (95%CI, 24.2–35.6)]. On the other hand, few participants touched the flat pad [*n* = 3, 1.2% (95%CI, 0.4–3.7). The top reasons for needed assistance (95%CI) was to read results [*n* = 40, 16.0% (11.0-21.1)], to place the tube into the plastic stand [*n* = 34, 13.8% (10.0-18.7) and to collect oral fluid sample [*n* = 20, 8.0% (5.2–12.1)]. A total of 72 participants [28.8% (95%CI, 23.5–34.8)] requested assistance at least for one step of oral fluid HCVST (Table 2). Additionally, five participants [2.0% (95%CI 0.8–4.8)] answered: “I did not know” for the question about oral fluid HCV self-test result.

### Factors Associated with Need of Assistance for HCVST

Table 3 describes factors associated with request of assistance for at least one step of blood-base and oral fluid HCVST [logistic regression analyses expressed as crude and adjusted-OR (95%CI)]. Education level lower than college degree (< 10 years) was independently associated with requesting assistance for at least one step of blood-based HCVST [aOR = 2.07 (95%CI, 1.99–3.59), *p* = 0.009]. Additionally, people who had at least one episode of chemsex in the last 6 months had a trend to not request assistance for HCVST using the blood-based kit [aOR = 0.55 (95%CI, 0.30–1.01), *p* = 0.053]. A high number of sexual partners (*n* > 10) [aOR = 2.00 (95%CI, 1.11–3.59), *p* = 0.020] and no condomless sexual intercourses [aOR = 2.75 (1.30–5.80), *p* = 0.008] in the last 6 months were significantly associated with need of assistance for oral fluid HCVST. On the other hand, lower education level was not associated with need of assistance for oral fluid HCVST [OR = 1.44 (95%CI 0.82–2.54)]. Supplementary Table 1 (S1 Table) summarizes assessment of difficulties, errors and necessity of assistance for blood-based and oral fluid HCVST stratified by schooling (people with or without college degree).

**Table 3:** Factors associated with request of assistance for at least one step of blood-base and oral fluid HCVST (*n* = 250)

	Blood-based HCV self-testing				Oral fluid HCV self-testing			
	Univariate analysis		Multivariate Model		Univariate analysis		Multivariate Model	
	Crude OR (95% CI)	*p* value	aOR (95% CI)	*p* value	Crude OR (95% CI)	*p* value	aOR (95% CI)	*p* value
Transgender women	1.86 (0.80–4.35)	0.152			1.96 (0.90–4.24)	0.088	1.59 (0.71–3.55)	0.263
Age > 30 years-old	1.20 (0.70–2.06)	0.504			1.33 (0.73–2.41)	0.346		
Black/Brown skin colour	1.60 (0.93–2.75)	0.087	1.33 (0.75–2.35)	0.328	1.27 (0.70–2.30)	0.437		
Family monthly income < 1 minimum wage	1.24 (0.71–2.17)	0.448			0.85 (0.47–1.55)	0.599		
Education level lower than college degree	2.34 (1.39–3.95)***	**0.001**	2.07 (1.99–3.59)**	**0.009**	1.44 (0.82–2.54)	0.202		
First time testing for HCV	1.18 (0.70–1.99)	0.532			1.14 (0.65–1.98)	0.645		
More than 10 sexual partners in the last 6 months	0.67 (0.40–1.29)	0.134			1.94 (1.10–3.40)*	**0.022**	2.00 (1.11–3.59)*	**0.020**
People who used condom in all sexual intercourses in the las 6 months	2.35 (1.02–1.96)*	**0.044**	1.73 (0.73–4.09)	0.215	2.59 (1.26–5.32)**	**0.010**	2.75 (1.30–5.80)**	**0.008**
People who had at least one episode of chemsex in the last 6 months	0.51 (0.28–0.91)*	**0.023**	0.55 (0.30–1.01)	0.053	1.01 (0.54–1.90)	0.981		

*aOR* adjusted odds ratio; Logistic regression analyses (crude and adjusted odds-ratios)

Bolded p-value = significant at * *p* ≤ 0.05, **p ≤ 0.01, ***p ≤ 0.001

Variables found to be associated [p value ≤ 0.10] with the need of assistance for at least one step during HCVST were entered into multivariate models

We did not observed collinearity among variables entered into multivariate models: variance inflation factor (VIF) for all variables < 1.10

### Re-reading and re-testing Concordance

Re-reading concordance was high (agreement = 99.6% and Cohen’s kappa = 0.92) for blood-based HCVST reported in 246 participants, since 4 individuals (1.6%) could not report HCVST result. A single test was interpreted as positive by the participant but was read as negative by the healthcare worker. A total of 2 tests (0.8%) were considered as invalid. A high agreement (99.6%) and Cohen’s kappa = 0.89 were observed for blood-based HCVST re-testing concordance (*n* = 244) excluding invalid tests. Regarding oral fluid HCVST, 100% re-reading agreement (with Cohen’s kappa = 1.00) was observed in 245 individuals [5 participants (2%) could not report results]. Additionally, 5 tests were considered as invalid (2%). Therefore, re-testing concordance was performed in 240 participants: agreement = 99.2% and Cohen’s kappa = 0.75 (SD = 0.06). Table 4 describes re-reading and re-testing agreements for blood-based and oral fluid HCVST. For blood-based HCVST, re-reading (Cohen’s kappa = 0.89 vs. 0.89) and re-testing agreement (Cohen’s kappa = 0.80 vs. 1.00) was slight lower in people without college degree compared to those with college degree (S2 Table). We observed re-reading agreement (Cohen’s kappa = 1.00), but a decrease in re-testing agreement (Cohen’s kappa = 0.50 vs. 1.00) in people with lower schooling (without college degree) compared to those with college degree for oral fluid HCVST (S3 Table).

**Table 4:** Assessment of re-reading and re-testing concordance for blood-based and oral fluid HCVST (*n* = 250)

Participant assessment	Re-reading by healthcare worker §			
	Negative	Positive	Invalid	Total
*Blood-based HCVST (n = 250)*				
Negative	239	0	0	239
Positive	1	4	0	4
Invalid	0	0	2	2
Total	240	4	2	246
Re-reading agreement = 99.6%; Cohen’s kappa = 0.92 (SD = 0.05)				

Participant assessment	Re-testing by healthcare worker ^¥^			
	Negative	Positive	Invalid	Total
Negative	239	0	-	239
Positive	1	4	-	5
Total	240	4	-	244
Re-testing agreement = 99.6%; Cohen’s kappa = 0.89 (SD = 0.06)				

Participant assessment	Re-reading by healthcare worker §			
	Negative	Positive	Invalid	Total
*Oral fluid HCVST (n = 250)*				
Negative	235	0	0	235
Positive	0	5	0	5
Invalid	0	0	5	5
Total	235	5	5	245
Re-reading agreement = 100%; Cohen’s kappa = 1.00				

Participant assessment	Re-testing by healthcare worker ^¥^			
	Negative	Positive	Invalid	Total
Negative	235	0	-	235
Positive	2	3	-	5
Total	237	3	-	240

Re-testing agreement = 99.2%; Cohen’s kappa = 0.75 (SD = 0.06)

Results were reported as absolute number (n)

Agreements were reported as proportions (%) and Cohen’s kappa index

§ The results of the HCV self-tests were reported by participants and re-assessed by a healthcare worker

¥ The results of the HCVST reported by participants were compared to the results of HCV test performed by a healthcare worker using a similar one-use-only kit excluding any invalid results

Blood-based HCVST (Wondfo HCV Self-Test^®^): (1) a total of 4 participants [1.6% (95%CI 0.6–4.2)] answered: “I did not know” for the question about HCV self-test result; (2) there were 2 invalid tests according to the participant’s assessment – analysis in 246 participants for re-reading and in 244 participants for re-testing analyses

The two tests reported as “invalid’ by the participant were re-assessed as “invalid” by the healthcare worker, but both were “negative” in re-testing

Oral fluid HCVST (OraQuick Hepatitis C Self-Test^®^): (1) a total of 5 participants [2.0% (95%CI 0.8–4.8)] answered: “I did not know” for the question about HCV self-test result; (2) there were 5 invalid tests according to the participant’s assessment – analysis in 240 participants for re-reading and for re-testing analyses

The five tests reported as “invalid’ by the participant were re-assessed as “invalid” by the healthcare worker, but four were “negative” and one was “positive” in re-testing

A total of 6 participants (2.4%) were referred for HCV-RNA due to positive result in at least one HCV test. Of them, five participants (83%) performed HCV-RNA test where 75% (*n* = 4) had detectable HCV viral load. All participants with detectable HCV-RNA were linked to treatment.

### Post-testing Perspectives

**Fig. 1:**
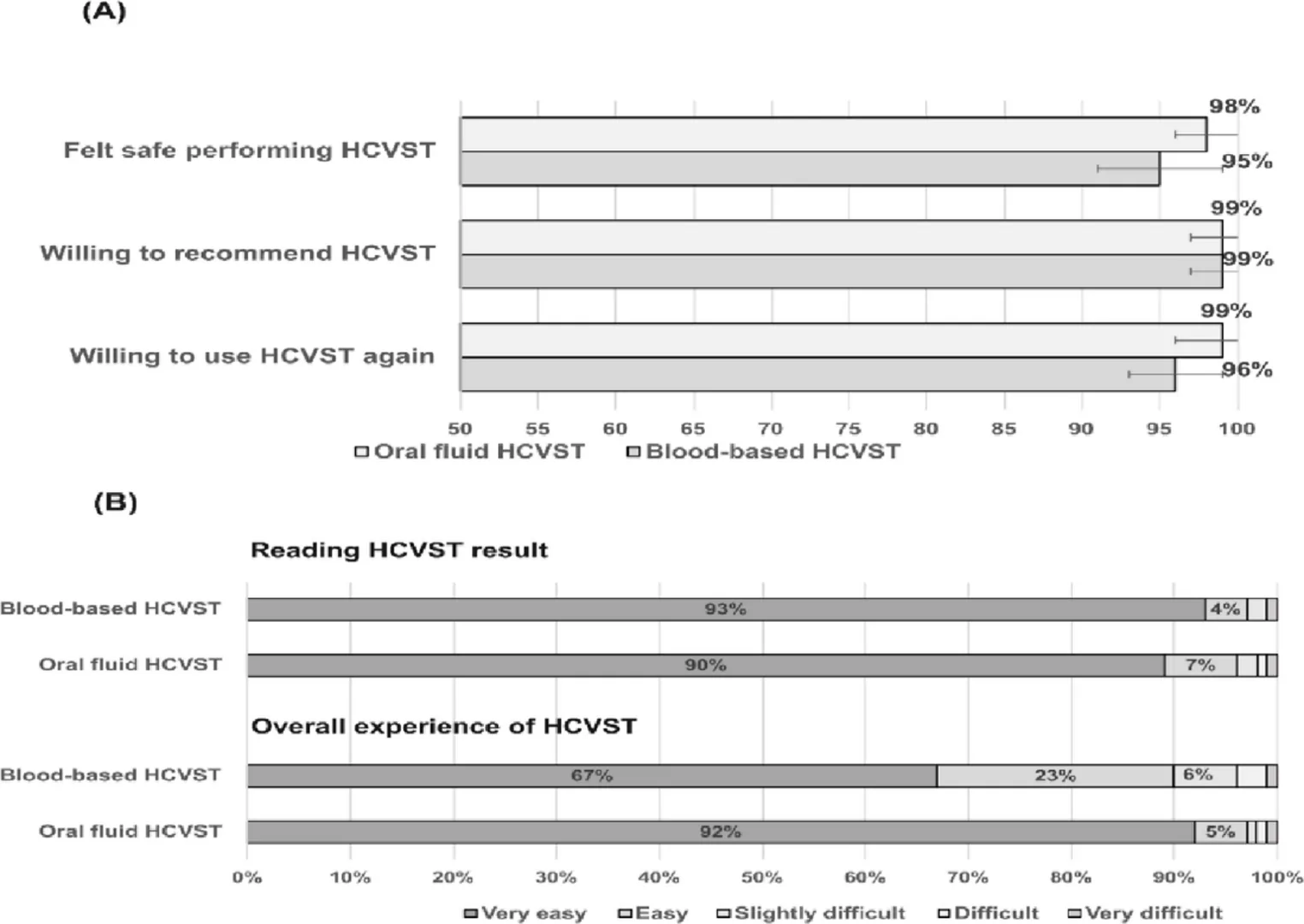
**A** Participant’s perspectives post-testing with oral fluid and blood-based HCV self-test kits and **B** perception of difficult for reading results and for overall experience to perform oral fluid and blood-based HCV self-testing. Oral fluid HCV self-test kit: OraQuick Hepatitis C Self-Test^®^ (OraSure Technologies, Bethlehem, USA)]; Blood-based HCV self-test kit: Wondfo HCV Self-Test^®^ (Wondfo Biotech Co, Guangzhou, China)

Table 5 describes preferences after performing both types of HCVST [expressed as N [% 95%CI)]. A total of 46.4% (*n* = 116) would prefer oral fluid, 36.4% (*n* = 91) would prefer blood-based and 17.2% (*n* = 43) had no preference between both HCV self-test types. Additionally, the top three preferences for performing future HCVST would be at home with IFU and step-by-step video (*n* = 140, 56.0%), at home with on-line support (*n* = 41, 16.4%) and at a facility-based with HCV testing performed by a healthcare worker (*n* = 26, 10.4%). People with higher education level were more likely to prefer oral fluid HCVST rather than blood-based test (Chi-Squared, *p* = 0.075) and were more likely to perform HCVST at home with IFU and video (Chi-Squared, *p* = 0.065). Most people felt safe performing oral fluid [98% (95%CI 96–99)] and blood-based HCVST [95% (95%CI 91–97)]. Additionally, most participants would be willing to recommend oral fluid [99% (95%CI 97–100)] or blood-based HCVST [99% (95%CI 97–100)] for a parent, a friend, or a sexual partner and would be willing to repeat oral fluid [99% (95%CI 96–100)] or blood-based HCVST [96% (95%CI 93–98)] (Fig. 1A). Most participants reported that reading HCVST results were very easy with blood-based [93% (95%CI 90–96)] or oral fluid kit [90% (95%CI 86–93)]. Considering the overall experience of HCVST, 67% (95%CI 61–72) considered blood-based HCVST as very easy. On the other hand, 92% (95%CI 88–95) reported the overall experience of oral fluid HCVST as very easy (Fig. 1B).

**Table 5:** Preferences of participants assessed after HCV self-testing using blood-based and oral fluid HCVST kits on the same day (*n* = 250)

Preferences post-testing	All § (*n* = 250) *N* [% (95%CI)]	People with a college degree ¥ (*n* = 106) *N* (%)	People without a college degree ¥ (*n* = 144) *N* (%)	*P* value
*Which type of HCV self-test kit did you prefer?* ^a^				0.075
Oral fluid HCV self-test	116 [46.4 (40.3–52.6)]	58 (54.7)	58 (40.3)	
Blood-based HCV self-test	91 [36.4 (30.6–42.6)]	32 (30.2)	59 (41.0)	
No preference between oral fluid or blood-based HCV self-test	43 [17.2 (13.0-22.4)]	16 (15.1)	27 (18.7)	
*Where would you like to perform HCVST in the future?* ^a^				0.065
At home with IFU and step-by-step video	140 [56.0 (49.8–62.1)]	69 (65.1)	71 (49.3)	
At home with on-line support	41 [16.4 (12.3–21.5)]	16 (15.1)	25 (17.4)	
At a facility-based scenario performed by an HCW	26 [10.4 (7.2–14.9)]	6 (5.7)	20 (13.9)	
At home with a friend/partner/family	13 [5.2 (3.0-8.8)]	3 (2.8)	10 (6.9)	
At home with a hotline support	10 [4.0 (2.2–7.3)]	5 (4.7)	5 (3.5)	
At a facility-based scenario as HCVST	7 [2.8 (1.3–5.8]	1 (0.9)	6 (4.2)	
At a community-center	3 [1.2 (0.4–3.7]	2 (1.9)	1 (0.7)	
At a pharmacy performed by an HCW	2 [0.8 (0.2–3.2)]	2 (1.9)	0 (0.0)	
No preference	7 [2.8 (1.3–5.8]	2 (1.9)	5 (3.5)	
Other	1 [0.4 (0.1–2.8)]	0 (0.0)	1 (0.7)	

§ Data expressed as absolute number (n) and proportion (%) (95% Confidence Interval, CI)

¥ Data was expressed as absolute number (n) and proportion (%)

P values was assessed by Chi-Square test

*IFU* instructions for use, *HCVST* HCV self-testing, *HCW* healthcare worker

## Discussion

This study highlighted the usability and re-reading/re-testing agreement of blood-based and oral fluid HCV self-test kits in cis-MSM and TGW at a PrEP clinic in a tertiary center in Rio de Janeiro (Brazil). We observed a higher proportion of people requesting assistance for blood-based (62.4%) compared to oral fluid HCVST (28.8%). Despite difficulties and errors during HCVST, this study reported a high re-reading and re-testing agreements. Finally, both HCV self-test kits were well accepted for HCV testing by this sample of participants. These findings support that HCVST could be integrated to PrEP services as an alternative for HCV testing, especially in a strategy combining digital approach along with medical expertise for prevention of HIV infection. Integrating HCVST into routine PrEP follow-up visits could optimize HCV screening aligned with HIV prevention care by using existing infrastructure.

The use of oral fluid self-test is feasible, quick, well-accepted, and might be a useful strategy to reach people at high risk for HCV infection, such as MSM [14]. Studies with limited sample size have described the feasibility of oral fluid HCVST in MSM at Vietnam [15]; China [16]; and Georgia [17]. Pooled data from those studies reported in a systematic review and meta-analysis (3 studies including 294 MSM) described that 90.8% (95%CI 85.9–94.8) correctly collected oral fluid sample and 78.1% (95%CI 53.4–95.3) performed oral fluid HCVST without needing assistance in any step [10]. Similarly, our study data showed that 71.2% of participants did not request assistance during oral fluid HCVST in the present study. On the other hand, 29.6% of our participants made errors in collecting oral fluid samples. Recently, we observed similar high proportion of incorrect oral fluid sample collection (32.8%) and high proportion of assistance requests for at least one step for oral fluid HCVST (35.6%) in 685 health-facility users in a single center of the Brazilian Primary Care System [18]. In the present study, we observed that people with a high number of sexual partners and those with no condomless sex in the last 6 months were more likely to request assistance for oral fluid HCVST. This may reflect a “motivated accuracy” approach, suggesting that individuals with higher partner counts or consistent condom use seek support to ensure procedural correctness and result validity.

Few studies have assessed usability of blood-based HCV self-test kits. Firstly, Majam et al. reported high feasibility and correctly interpretation of results of three HCVST fingerstick prototypes in young adults, 30% with high education levels, from the general population of South Africa [19]. More recently, usability and acceptability of blood based (First Response, Premier Medical Corporation, India) and oral fluid HCVST (OraQuick, OraSure Technologies, USA) performed on the same day were assessed in 100 MSM from Malaysia [20]. Comparable to our study, they included young adults (median age = 30 years) with high education level. Few people requested assistance for HCVST using oral fluid (6%) or blood-based (10%) HCV self-test kits. On the other hand, at least one error was observed during oral fluid HCVST in 47% of participants using an HCV self-test kit from the same manufacturer used in the present study. Additionally, similar to our findings, those authors also observed a high proportion of people who made at least one error during blood-based HCVST (70%), despite using a different blood-based HCV self-test kit from our study.

The relatively high proportion of errors and/or need of assistance might be a potential issue for self-testing. In a study including 295 participants in a high HCV-seroprevalence population living in Pakistan, where 32% were illiterate, 23% (*n* = 67) needed assistance in one step and 64% (*n* = 190) in two or more steps for oral fluid HCVST (OraQuick, OraSure Technologies, USA) [21]. In the present study, requesting assistance was strongly associated with lower education level for blood-based, but not for oral fluid self-test. This raises the question whether HCVST, especially using a blood-based self-test, might be implemented as an assisted-test rather than self-testing. Major challenges observed in the present study with the blood-based HCVST were to obtain blood sample with the plastic dropper (34% requested assistance for this step), as well as to add blood (31%) or buffer in the well (36%) in the test device. Cultural differences might influence the proportion of people requiring assistance to performing self-testing, across different regions/countries. Further studies assessing blood-based HCVST with and without assistance would be needed. Despite those errors or need of assistance, we reported high agreement for reading results. These findings are similar to those reported in the studies conducted in Malaysia (> 95% of inter-operator concordance for both kits) [20] and in Pakistan (inter-reader concordance = 96% and Cohen’s kappa = 0.89 for oral fluid HCV self-test) [21]. Overall, most of the test results were interpreted correctly by MSM using oral fluid and blood-based kits in all studies.

Post-testing acceptability was high across all settings. In our Brazilian cohort, over 95% of participants were willing to repeat or recommend HCVST, a finding consistent with previous studies in Malaysia and Pakistan. When comparing preferences for specific HCVST types, Brazilian participants showed a slight preference for oral fluid (46%) over blood-based kits (36%), which would be similar to the results from Malaysia (44% oral fluid vs. 31% blood-based). In contrast, participants in Pakistan strongly preferred oral fluid kits (70% vs. 30% for blood-based). Preferences regarding the preferred setting for future testing also varied by region. While participants in Malaysia preferred supervised self-testing at primary care units (38%) or home-based testing (16%), our Brazilian cohort predominantly favored home-based testing, either independently (56%) or with online support (16%), with only a small fraction preferring facility-based testing (~ 3%). This strong preference for self-testing outside clinical settings aligns with findings from Pakistan, where 96% of participants favored HCVST over clinic-based testing.

This study has limitations. A convenience sample of people attending a PrEP consultation (initial or follow-up) in a tertiary center specialized in HIV care (INI/Fiocruz) were invited to participate. People enrolled in the study might be those more concerned about their healthcare. We acknowledge that this have led to a selection bias, then this sample might not be representative of people using PrEP in Brazil. Difficulties to collect samples and/or to interpret IFU can be barriers/challenges for HCVST [22]. People on PrEP are relatively used to perform HIV self-testing. Moreover, we used IFUs provided by the manufacturers that were translated to Portuguese, printed with high-quality paper and included in each test pouch. We are aware that people may have felt embarrassed by the presence of the healthcare worker, and this could have impacted our findings regarding the proportion of errors during self-testing. Additionally, as a cross-sectional study assessing first-time HCVST use, our findings may overestimate usability challenges, since proficiency likely improves with repeated testing. Further studies might be needed to provide evidence of feasibility of unsupervised HCVST and to better characterize learning curves of HCVST. The main strength of this study was the use of a WHO pre-qualified oral fluid HCVST kit (OraQuick HCV Self-Testing). Additionally, we generate evidence of feasibility of two kits (oral fluid and blood-based) for HCVST among a relatively large sample of MSM and TGW on PrEP in a Western country. Moreover, HCVST procedures were observed by a single trained nurse using structured questionnaires/checklists.

In summary, oral fluid and blood-based HCVST was feasible and well-accepted among people on PrEP (88% MSM) in Rio de Janeiro (Brazil). However, the relatively high proportion of individuals requiring assistance, especially for blood-based self-testing, can pose significant barriers to implement HCVST in real-world settings. Despite observed usability challenges, the high re-reading and re-testing agreement supports HCVST as a highly accurate tool that could significantly expand testing coverage in high-prevalence settings. Future research, especially to assess unsupervised testing, best ways to distribute kits, and cost-effectiveness analyses considering local/regional context are needed to support the use of HCVST as a public health strategy. Additionally, an affordable price of self-tests kits and availability of linking to virologic testing for confirming HCV diagnosis prior to treatment remains a major obstacle to HCV elimination and must be urgently addressed.

## Supplementary Information

Below is the link to the electronic supplementary material.

Supplementary Material 1

## Data Availability

All data from the current study were based on published studies and reported in the manuscript, tables and supplementary material. In addition, data are available upon a reasonable request to the corresponding author, from Evandro Chagas National Institute of Infectious Diseases (INI), Oswaldo Cruz Foundation (FIOCRUZ), Rio de Janeiro (RJ), Brazil.

## Abbreviations

aOR: Adjusted odds ratio
CI: Confidence interval
ERC: Ethical review committee
HCV: Hepatitis C virus
HCVST: HCV self-testing
IFU: Instructions for use
INI/FIOCRUZ: Evandro Chagas national institute of infectious diseases from the Oswaldo Cruz foundation
IQR: interquartile range
IRB: Institutional Review Board
REDCap: Research electronic data capture
MSM: Men who have sex with men
PrEP: Pre-exposure prophylaxis
RUO: Research use only
TGW: Transgender women
VIF: Variance inflation factor
WHO: World Health Organization

## References

[1] World Health Organization. Hepatitis C. Hepatitis C [https://www.who.int/news-room/fact-sheets/detail/hepatitis-c. ].

[2] Jin F, Dore GJ, Matthews G, Luhmann N, Macdonald V, Bajis S, et al. Prevalence and incidence of hepatitis C virus infection in men who have sex with men: a systematic review and meta-analysis. Lancet Gastroenterol Hepatol. 2021;6(1):39–56. 33217341 10.1016/S2468-1253(20)30303-4

[3] Gerk A, Telles L, Carroll M, Nascimento M, Bispo RG, Oliveira BFS, et al. Use of industrial liquid silicone: a scoping review. Acta Cir Bras. 2024;39:e395624. 39383418 10.1590/acb395624 PMC11457952

[4] HCV Polaris Observatory. Global change in hepatitis C virus prevalence and cascade of care between 2015 and 2020: a modelling study. Lancet Gastroenterol Hepatol. 2022;7(5):396–415. 35180382 10.1016/S2468-1253(21)00472-6

[5] Grebely J. Point-of-Care Testing for Hepatitis C Infection: A Critical Building Block for the Foundation to Achieve Elimination. Clin Infect Dis. 2024;79(4):974–7. 38513081 10.1093/cid/ciae156

[6] Barter L, Cooper CL. The impact of electronic medical record system implementation on HCV screening and continuum of care: a systematic review. Ann Hepatol. 2021;24:100322. 33549734 10.1016/j.aohep.2021.100322

[7] O’Keefe D, Jacka D, Douglass C, Gunn J, Stoove M, Crawford S, et al. Distribution of rapid HCV antibody self-test kits via needle/syringe dispensing machines: Implementation and evaluation of the Vend-C pilot study in Melbourne, Australia. J Viral Hepat. 2024;31(3):151–5. 38158743 10.1111/jvh.13909

[8] Venkatesan P. First self-test for hepatitis C virus. Lancet Microbe. 2024;5(12):100979. 39241796 10.1016/j.lanmic.2024.100979

[9] Shin G, Kim BK, Bae S, Lee H, Ahn SH. Self-testing strategy to eliminate hepatitis C as per World Health Organization’s goal: Analysis of disease burden and cost-effectiveness. Clin Mol Hepatol. 2025;31(1):166–78. 39363405 10.3350/cmh.2024.0484 PMC11791605

[10] Perazzo H, Castro R, Villela-Nogueira C, Torres M, Silva SL, Cardoso SW, et al. Acceptability and usability of oral fluid HCV self-testing for hepatitis C diagnosis: A systematic review and meta-analysis. J Viral Hepat. 2023;30(11):838–47. 37485619 10.1111/jvh.13876

[11] Melnychuk S, Balakireva O, Pavlova D, Lopatenko A, McClarty LM, Lazarus L, et al. Joint HIV and hepatitis C virus phylogenetic analyses signal network overlap among women engaged in sex work and men who purchase sex. Int J STD AIDS. 2025;36(7):542–9. 39325924 10.1177/09564624241287259 PMC12085746

[12] Gil Minana J, Arias Garcia S, De Lazzari E, Montoya Conesa FJ, De La Mora L, Laguno M, et al. Chemsex and uptake of HIV pre-exposure prophylaxis among adult cisgender gay, bisexual and other men who have sex with men worldwide: a systematic review with meta-analysis. EClinicalMedicine. 2026;93:103804. 41938845 10.1016/j.eclinm.2026.103804 PMC13043317

[13] Harris PA, Taylor R, Thielke R, Payne J, Gonzalez N, Conde JG. Research electronic data capture (REDCap)--a metadata-driven methodology and workflow process for providing translational research informatics support. J Biomed Inf. 2009;42(2):377–81. 10.1016/j.jbi.2008.08.010 PMC2700030 18929686

[14] Albertos S, Majo FX, Esteban R, Colom J, Buti M. A large-scale screening of hepatitis C among men who have sex with men in the community using saliva point-of-care testing. Front Public Health. 2024;12:1478195. 39717035 10.3389/fpubh.2024.1478195 PMC11663926

[15] Nguyen LT, Nguyen VTT, Le Ai KA, Truong MB, Tran TTM, Jamil MS et al. Acceptability and Usability of HCV Self-Testing in High Risk Populations in Vietnam. Diagnostics (Basel) 2021, 11(2). 10.3390/diagnostics11020377 PMC7926709 33672241

[16] Xu W, Reipold EI, Zhao P, Tang W, Tucker JD, Ong JJ, et al. HCV Self-Testing to Expand Testing: A Pilot Among Men Who Have Sex With Men in China. Front Public Health. 2022;10:903747. 35712303 10.3389/fpubh.2022.903747 PMC9194083

[17] Fajardo E, Watson V, Kumwenda M, Usharidze D, Gogochashvili S, Kakhaberi D, et al. Usability and acceptability of oral-based HCV self-testing among key populations: a mixed-methods evaluation in Tbilisi, Georgia. BMC Infect Dis. 2022;22(1):510. 35641908 10.1186/s12879-022-07484-2 PMC9154030

[18] Perazzo H, Villela-Nogueira C, Gomes MK, Daher A, Siqueira-do-Valle C, Zukeram K, et al. Acceptability and usability of oral fluid HCV self-testing among health-facility users from Brazil: a cross-sectional study of 685 participants. Braz J Infect Dis. 2025;29(4):104544. 40412028 10.1016/j.bjid.2025.104544 PMC12151183

[19] Majam M, Fischer A, Ivanova Reipold E, Rhagnath N, Msolomba V, Lalla-Edward ST. A Lay-User Assessment of Hepatitis C Virus Self-Testing Device Usability and Interpretation in Johannesburg, South Africa. Diagnostics (Basel) 2021, 11(3). 10.3390/diagnostics11030463 PMC8000311 33800060

[20] Chan HK, Sem X, Ivanova Reipold E, Pannir Selvam SBA, Salleh NA, Mohamad Gani AHB, et al. Usability and acceptability of oral fluid- and blood-based hepatitis C virus self-testing among the general population and men who have sex with men in Malaysia. PLOS Glob Public Health. 2024;4(1):e0001770. 38170720 10.1371/journal.pgph.0001770 PMC10763960

[21] Mazzilli S, Aslam MK, Akhtar J, Miazek M, Wailly Y, Hamid S, et al. Usability and acceptability of self-testing for hepatitis C virus exposure in a high-prevalence urban informal settlement in Karachi, Pakistan. BMC Infect Dis. 2024;24(1):1054. 39333922 10.1186/s12879-024-09925-6 PMC11428378

[22] Borek AJ, Roleston C, Lazzarino R, Cooray M, Hayward G, Roberts N, et al. Acceptability of self-sampling and self-testing for infections: a rapid systematic review on public users’ views. BMC Public Health. 2025;25(1):695. 39972444 10.1186/s12889-025-21773-w PMC11841015

