# Supplementary material for: Acceptability and Usability of Blood-Based and Oral Fluid HCV Self-Testing Among Men-Who-Have-Sex-with-Men and Transgender Women on PrEP
Source: AIDS Behav. 2026 Jun 22;30(9):2887–97. doi: 10.1007/s10461-026-05207-1 (PMC13586042; doi:10.1007/s10461-026-05207-1)
Supplement: Supplementary file 1 [file 10461_2026_5207_MOESM1_ESM.docx]

**Acceptability and usability of blood-based and oral fluid HCV self-testing among men-who-have-sex-with-men and transgender women on PrEP**

**SUPPLEMENTARY MATERIAL**

|  | **Page** |
| --- | --- |
| **S1 Table.** Healthcare provider assessment of difficulties, errors and necessity of assistance for blood-based and oral fluid HCV self-testing (HCVST) stratified by schooling | 1 |
| **S2 Table.** Sensitivity analyses of re-reading and re-testing concordance analyses according to education level (with vs without a college degree) for blood-based HCV self-testing (Wond-Fo^®^) | 2 |
| **S3 Table.** Sensitivity analyses of re-reading and re-testing concordance analyses according to education level (with vs without a college degree) for oral fluid HCV self-testing (OraQuick^®^) | 3 |

**S1 Table.** Healthcare provider assessment of difficulties, errors and necessity of assistance for blood-based and oral fluid HCV self-testing (HCVST) stratified by schooling

|  | **College degree or higher (n=106)** | **Less than college degree (n=144)** | **p value** |
| --- | --- | --- | --- |
| **Blood-based HCVST (n=250)** |  |  |  |
| **Pre-testing activities** |  |  |  |
| People who read the IFU | 54 (50.9) | 52 (36.1) | 0.019 |
| People who watched step-by-step video | 48 (45.3) | 78 (54.2) | 0.17 |
| **Difficulties observed during HCVST** |  |  |  |
| To fingerprick | 14 (13.2) | 40 (27.8) | 0.006 |
| To collect blood sample with the dropper | 35 (33.0) | 65 (45.1) | 0.053 |
| To add blood sample in the specimen well | 16 (15.1) | 35 (24.3) | 0.074 |
| **Errors observed during HCVST** |  |  |  |
| To add buffer in the buffer well | 13 (12.3) | 10 (6.9) | 0.15 |
| To timekeeping for reading results | 7 (6.6) | 15 (10.4) | 0.29 |
| **Assistance needed during HCVST** |  |  |  |
| To fingerprick | 13 (12.3) | 39 (27.1) | 0.004 |
| To collect blood sample with the dropper | 27 (25.5) | 58 (40.3) | 0.015 |
| To add blood sample in the specimen well | 33 (31.1) | 44 (30.6) | 0.92 |
| To add buffer in the buffer well | 33 (31.1) | 56 (38.9) | 0.21 |
| To read HCVST result | 15 (14.2) | 30 (20.8) | 0.17 |
| Assistance in at least one step of HCVST | 54 (50.9) | 102 (70.8) | 0.001 |
| Assistance needed in at least one step of HCVST without the use of dropper | 50 (47.2) | 92 (63.9) | 0.008 |
| **Oral fluid HCVST (n=250)** |  |  |  |
| **Pre-testing activities** |  |  |  |
| People who read the IFU | 58 (54.7) | 54 (37.5) | 0.007 |
| People who watched step-by-step video | 51 (48.1) | 79 (54.9) | 0.29 |
| **Difficulties observed during HCVST** |  |  |  |
| To insert test cassette in the tube | 5 (4.7) | 9 (6.2) | 0.60 |
| **Errors observed during HCVST** |  |  |  |
| Touched the paddle | 1 (0.9) | 2 (1.4) | 0.75 |
| To collect oral fluid sample | 30 (28.3) | 44 (30.6) | 0.70 |
| To timekeeping for reading results | 6 (5.7) | 15 (10.4) | 0.18 |
| **Assistance needed during HCVST** |  |  |  |
| To open the tube | 5 (4.7) | 13 (9.0) | 0.19 |
| To place the tube into the plastic stand | 13 (12.3) | 21 (14.6) | 0.55 |
| To collect oral fluid sample | 7 (6.6) | 13 (9.0) | 0.49 |
| To place the test device in the test tube | 4 (3.8) | 7 (4.9) | 0.68 |
| To read HCVST result | 14 (13.2) | 26 (18.1) | 0.30 |
| Assistance in at least one step of HCVST | 26 (24.5) | 46 (31.9) | 0.17 |

HCVST, hepatitis C virus self-testing; IFU, instructions for use

**S1 Table.** Sensitivity analyses of re-reading and re-testing concordance analyses according to education level (with vs without a college degree) for blood-based HCV self-testing [Wondfo HCV Self-Test® (Wondfo Biotech Co, Guangzhou, China)]

| **Re-reading concordance analysis §** | | | | |
| --- | --- | --- | --- | --- |
|  | **People with a college degree (n=104)** | | | |
| **Participant assessment** | **Negative** | **Positive** | **Invalid** | **Total** |
| **Negative** | 102 | 0 | 0 | 102 |
| **Positive** | 0 | 2 | 0 | 2 |
| **Invalid** | 0 | 0 | 0 | 0 |
| **Total** | 102 | 2 | 0 | 104 |
| **Re-reading agreement=100%; Cohen's kappa=1.00 (SD=0.10)** | | | | |
|  | **People without a college degree (n=142)** | | | |
| **Participant assessment** | **Negative** | **Positive** | **Invalid** | **Total** |
| **Negative** | 137 | 0 | 0 | 137 |
| **Positive** | 1 | 2 | 0 | 3 |
| **Invalid** | 0 | 0 | 2 | 2 |
| **Total** | 138 | 2 | 2 | 142 |
| **Re-reading agreement=99.3%; Cohen's kappa=0.89 (SD=0.07)** | | | | |
| **Re-resting concordance analysis (excluding invalid tests) ¥** | | | | |
|  | **People with a college degree (n=104)** | | | |
| **Participant assessment** | **Negative** | **Positive** | **Invalid** | **Total** |
| **Negative** | 102 | 0 | - | 102 |
| **Positive** | 0 | 2 | - | 2 |
| **Total** | 102 | 2 | - | 104 |
| **Re-reading agreement=100%; Cohen's kappa=1.00 (SD=0.10)** | | | | |
|  | **People without a college degree (n=140)** | | | |
| **Participant assessment** | **Negative** | **Positive** | **Invalid** | **Total** |
| **Negative** | 137 | 0 | - | 137 |
| **Positive** | 1 | 2 | - | 3 |
| **Total** | 138 | 2 | - | 140 |
| **Re-reading agreement=99.3%; Cohen's kappa=0.80 (SD=0.08)** | | | | |

§ The results of the HCV self-tests were reported by participants and re-assessed by a healthcare worker. This analysis was performed in 246 participants because 4 participants [1.6% (95%CI 0.6-4.2)] answered: “I did not know” for the question about HCV self-test result [50% (n=2) by without a college degree]. ¥ The results of the HCVST reported by participants were compared to the results of HCV test performed by a healthcare worker using a similar one-use-only kit excluding any invalid results. This analysis was performed in 244 participants because 4 participants [1.6% (95%CI 0.6-4.2)] answered: “I did not know” for the question about HCV self-test result [50% (n=2) by without a college degree] and 2 participants reported HCVST result as “invalid” (excluded from this analysis).These 2 invalid tests according to the participant’s assessment [100% (n=2) by without a college degree] – both were reported as “invalid” when checked by the HCW, but they were “negative” by re-testing with a similar HCV kit.

**S3 Table.** Sensitivity analyses of re-reading and re-testing concordance analyses according to education level (with vs without a college degree) for oral fluid HCV self-testing [OraQuick Hepatitis C Self-Test® (OraSure Technologies, Bethlehem, USA)]

| **Re-reading concordance analysis §** | | | | |
| --- | --- | --- | --- | --- |
|  | **People with a college degree (n=103)** | | | |
| **Participant assessment** | **Negative** | **Positive** | **Invalid** | **Total** |
| **Negative** | 97 | 0 | 0 | 97 |
| **Positive** | 0 | 2 | 0 | 2 |
| **Invalid** | 0 | 0 | 4 | 4 |
| **Total** | 97 | 2 | 4 | 103 |
| **Re-reading agreement=100%; Cohen's kappa= 1.00 (SD=0.08)** | | | | |
|  | **People without a college degree (n=142)** | | | |
| **Participant assessment** | **Negative** | **Positive** | **Invalid** | **Total** |
| **Negative** | 138 | 0 | 0 | 138 |
| **Positive** | 0 | 3 | 0 | 3 |
| **Invalid** | 0 | 0 | 1 | 1 |
| **Total** | 138 | 3 | 1 | 142 |
| **Re-reading agreement=100%; Cohen's kappa=1.00 (SD=0.07)** | | | | |
| **Re-resting concordance analysis (excluding invalid tests) ¥** | | | | |
|  | **People with a college degree (n=104)** | | | |
| **Participant assessment** | **Negative** | **Positive** | **Invalid** | **Total** |
| **Negative** | 97 | 0 | - | 97 |
| **Positive** | 0 | 2 | - | 2 |
| **Total** | 97 | 2 | - | 99 |
| **Re-reading agreement=100%; Cohen's kappa= 1.00 (SD=0.10)** | | | | |
|  | **People without a college degree (n=140)** | | | |
| **Participant assessment** | **Negative** | **Positive** | **Invalid** | **Total** |
| **Negative** | 138 | 0 | - | 138 |
| **Positive** | 2 | 1 | - | 3 |
| **Total** | 140 | 1 | - | 141 |
| **Re-reading agreement=98.6%; Cohen's kappa=0.50 (SD=0.07)** | | | | |

§ The results of the HCV self-tests were reported by participants and re-assessed by a healthcare worker. This analysis was performed in 245 participants because 5 participants [2.0% (95%CI 0.8-4.8)] answered: “I did not know” for the question about HCV self-test result [40% (n=2) by people without a college degree]. ¥ The results of the HCVST reported by participants were compared to the results of HCV test performed by a healthcare worker using a similar one-use-only kit excluding any invalid results. This analysis was performed in 240 participants because 5 participants [2.0% (95%CI 0.8-4.8)] answered: “I did not know” for the question about HCV self-test result [40% (n=2) by people without a college degree. A total of 5 participants reported HCVST result as “invalid” (excluded from this analysis).These 5 invalid tests according to the participant’s assessment [80% (n=4) by people with a college degree] – all were reported as “invalid” when checked by the HCW, but four were “negative” and one was “positive” by re-testing with a similar HCV kit.
